# Relationship Between Cardiovascular Disease Risk and Long-Term Neurological Sequelae After Carbon Monoxide Poisoning: A Nationwide Cohort Study

**DOI:** 10.3390/jcm15062338

**Published:** 2026-03-18

**Authors:** Min-Po Ho, Yuan-Hui Wu, Tsan-Chi Chen, Kuang-Chau Tsai, Chen-Chang Yang, Feng-Yuan Chu

**Affiliations:** 1 Department of Emergency Medicine, Far Eastern Memorial Hospital, New Taipei City 220, Taiwan; hominpo@yahoo.com.tw (M.-P.H.); wuyuanhui@ntu.edu.tw (Y.-H.W.); hikali@mail.femh.org.tw (K.-C.T.); 2 School of Medicine, Fu-Jen Catholic University, New Taipei City 242, Taiwan; 3 Department of Medical Research, Far Eastern Memorial Hospital, New Taipei City 220, Taiwan; 960887272@mail.femh.org.tw; 4 Institute of Public Health, National Yang Ming Chiao Tung University, Taipei 112, Taiwan; 5 Institute of Environmental and Occupational Health Sciences, National Yang Ming Chiao Tung University School of Medicine, 155, Section 2, Linong Street, Taipei 112, Taiwan; 6 Department of Occupational Medicine & Clinical Toxicology, Taipei Veterans General Hospital, Taipei 112, Taiwan; 7 School of Medicine, College of Medicine, National Yang Ming Chiao Tung University, Taipei 112, Taiwan

**Keywords:** carbon monoxide poisoning, cardiovascular disease, neurological sequelae, long term complications

## Abstract

**Background:** Carbon monoxide poisoning (COP) has emerged as a significant health issue in Asian countries, including Taiwan. It poses serious risks, including long-term complications such as cardiovascular disease (CVD), neurological disorders, and even death. This study investigated the association of COP with the development of cardiovascular diseases and neurological sequelae, while evaluating all-cause and cause-specific mortality as secondary outcomes. **Methods:** This retrospective study utilized the National Health Insurance Research Database and included the patients aged ≥ 20 years hospitalized with a COP diagnosis between 1 January 2000 and 31 December 2015. The objective was to investigate long-term neurological complications, CVD (such as ischemic heart disease and other cardiac conditions), and associated risk factors. Cox proportional hazard regression was employed to analyze differences in long-term neurological sequelae and cardiovascular outcomes among various groups. **Results:** A total of 2421 COP patients were enrolled. COP patients with CVD history had a higher incidence of persistent neurological sequelae (PNS) in two different diagnostic codes (8.6%, *p* < 0.001 and 11.5%, *p* = 0.018), but COP patients without CVD history had a higher incidence of delayed neurological sequelae (DNS) only in one of the diagnostic codes (6.8%, *p* < 0.001). The risk from CVD factor was up to 11.92 times. Furthermore, the overall mortality was 8.8%, which is significantly higher than 3.7% in the general population. After adjusting for other factors, the mortality in COP individuals was 7.40 times higher than that of the general population. **Conclusions:** Patients with COP might be at high risk of developing CVD and have a significantly increased risk of CVD. COP is associated with a higher risk of long-term neurological complications and an increased incidence of CVD. These findings help mitigate the potential long-term health impacts of COP.

## 1. Introduction

Carbon monoxide poisoning (COP) is a serious global health issue due to its severe clinical effects and high toxicity, morbidity, and mortality [1]. In the United States, COP also leads to approximately 50,000 emergency department visits and 2700 deaths annually [2]. In addition, COP survivors are at higher risk of long-term mortality [3], especially those who were intentionally exposed. Ventricular dysfunction, myocardial infarction, and dysrhythmia are often seen in patients with moderate to severe poisonings and are associated with higher mortality [3].

In an emergency, COP can affect organs that are particularly sensitive to low oxygen levels, such as heart and brain, and present with a variety of symptoms [4,5,6]. Both heart and brain are highly susceptible to hypoxia, which in severe cases of COP can lead to significant risks such as cardiovascular complications and delayed neurological issues [7]. More than 25% of patients with COP have cardiovascular problems, typically manifested by elevated cardiac biomarkers and/or electrocardiogram changes [8,9]. In general, exposure to carbon monoxide can cause physical symptoms such as headache, blurred vision, increased heart rate and breathing, unsteady gait, cognitive impairment, and fainting. Furthermore, severe exposure can lead to cardiac and respiratory depression, arrhythmias, rhabdomyolysis, renal failure, coma, pulmonary edema, seizures of epilepsy, and even death [6,10]. After recovery from emergency treatment, COP can lead to long-term neurological complications, including delayed neurological sequelae (DNS) and persistent neurological sequelae (PNS) [11,12,13]. Neurological complications may be due not only to the strong binding of carbon monoxide to hemoglobin, but also to inflammatory effects, membrane peroxidation, oxygen radicals, and ischemia–reperfusion injury [14]. These neurological complications affect 15–40% of COP survivors [6]. In addition, risk factors for cardiovascular disease (CVD), such as hypertension, diabetes, hyperlipidemia, smoking, and excessive alcohol consumption, may also contribute to higher mortality in COP patients [15]. Furthermore, CVD risk is frequently identified as a significant consequence of carbon monoxide exposure [3] and has been shown to predict DNS in affected patients [3,16].

This study primarily attempts to investigate the potential impact of COP on the development of cardiovascular diseases and neurological sequelae using the National Health Insurance Research Database (NHIRD). All-cause and cause-specific mortality were evaluated as secondary outcomes.

## 2. Materials and Methods

### 2.1. Data Source

This research was approved by the Human Research Ethics Committee of Taipei Veterans General Hospital (IRB No. 2017-07-018AC). The NHIRD analysis was primarily based on the Longitudinal Generation Tracking Database 2000 (LGTD 2000). The Health and Welfare Data Science Center of the Ministry of Health and Welfare provided medical records and cause of death data for two million people. The NHIRD encompasses data from Taiwan’s National Health Insurance Program, which covers nearly 100% of the population, including foreigners residing in the country. The NHIRD from the National Health Research Institutes (NHRI) provided research purposes for scientists in Taiwan.

### 2.2. Classification of COP and CVD

Since the launch of the NHIRD in 1995, the data were initially incomplete and became more comprehensive by 2000. Thus, in this study, more complete COP cases aged ≥ 20 years were enrolled between 1 January 2000 and 31 December 2015. Based on the inpatient claims, patients who were hospitalized for COP (ICD-9-CM code 986), with no medical history of arrhythmia (ICD-9-CM codes 427), coronary artery disease (ICD-9-CM codes 410–414), congestive heart failure (ICD-9-CM code 428), and complete age or sex information, were enrolled in the study. To reduce potential diagnostic or coding errors, only patients with the diagnoses, who received hyperbaric oxygen (HBO) treatment or 100% oxygen therapy during emergency visit, or were hospitalized for treatment after the emergency visit, were considered as COP cases. If interference factors are not fully controlled, they can lead to bias in the research results.

In the relevant statistical analysis, cases of concomitant exposure to specific highly toxic substances, primarily pesticides (ICD-9-CM codes 989.3–989.4), were excluded. This approach aims to minimize the potential impact of such poisoning on the research findings. COP cases were matched to the selected non-COP group, using a ratio of 1:4 based on age and gender, resulting in corresponsive individuals from the general population who had no history of poisoning, to do further analysis. This section delves into whether there are differences in the incidence of cardiovascular patients such as ischemic heart disease and other forms of cardiac disease (ICD-9-CM 410–414, 424–429) with COP over the long term (onset more than a year after a COP event) as the primary cardiovascular outcome.

To ensure the accuracy of the related diagnoses as much as possible, cases with at least three consecutive outpatient diagnoses or those hospitalized for associated diseases were selected. CVD patients within a year before the COP incident were excluded for a subgroup analysis, ensuring only those potentially having CVD risk after the poisoning were considered for the primary cardiovascular outcome analysis.

The mortality data from the Ministry of Health and Welfare from 2000 to 2015 were explored, whether the mortality rate of COP patients was indeed higher, and whether there were differences in CVD risk and major causes of death such as suicide, as secondary outcomes. To understand the potential impact of the severity of COP on long-term neurological complications, the research team used indicators like endotracheal intubation and admission to the intensive care unit as markers of severe COP. This approach partly addresses the limitations of the national health insurance dataset, which lacks laboratory data on carboxyhemoglobin levels and cardiac injury biomarkers (e.g., troponin), as well as other bedside measures of clinical severity.

### 2.3. Diagnosis of DNS and PNS

The occurrence of DNS or PNS as the primary neurological outcomes was defined according to the relevant ICD-9-CM codes and the time interval following COP. PNS was defined as an initial neurological dysfunction that persists up to six weeks after COP. DNS was characterized by the onset of neuropsychiatric symptoms occurring within three months after the completion of treatment for COP. Based on the time difference between these diagnoses and the COP incident, the identified patients might have developed DNS/PNS post-COP.

Follow-up began at the index date and continued until the outcome, death, disenrollment, or 31 December 2015. For incident CVD, individuals with CVD within one year prior to index were excluded; outcomes within the first year were not counted toward long-term incidence. DNS/PNS were assessed within their predefined windows.

### 2.4. Statistical Analysis

Primary outcomes were incident long-term CVD and DNS/PNS, and mortality outcomes were assessed secondarily. The chi-square and *t*-tests were used to compare demographic characteristics and medical histories between the COP group and the non-COP group. To adjust for differences in baseline health status between the two groups, a multivariate Cox proportional hazard model was employed to estimate the independent COP impact on the risk of long-term neurological complications, CVD, and mortality. Across all Cox analyses, deaths were handled as competing events: for cause-specific mortality outcomes, deaths from other causes ended follow-up, and for non-fatal outcomes, all-cause death ended follow-up. Several potential confounders were adjusted, including year of poisoning, age, gender, financial status, smoking habit, drinking behavior, HBO therapy, residence area, frequency of clinic visits, and various comorbidities such as diabetes, hypertension, hyperlipidemia, gout, CVD, chronic liver disease, chronic kidney disease, and mental illness. The goal was to identify differences in long-term outcomes such as neurological complications and CVD. The associated hazard ratios (HRs) and 95% confidence intervals (CIs) were calculated.

Primary outcomes were: (1) incident CVD (ICD-9-CM 410–414, 424–429) assessed > 1 year after index; and (2) neurological sequelae: PNS (initial neurological dysfunction persisting ≤ 6 weeks after COP) and DNS (new neuropsychiatric symptoms within 3 months after completion of COP treatment), each identified using two diagnostic code groups (group I: narrower; group II: broader). Secondary outcomes were all-cause mortality and cause-specific mortality (CVD and suicide) obtained from linked death certificates.

All statistical analyses were anticipated to be performed using SAS version 9.4, with a *p*-value of less than 0.05 considered statistically significant.

## 3. Results

### 3.1. General Characteristics of COP Patients

Between 2000 and 2015, 2421 cases with COP were identified from a total of 12,105 enrolled cases, excluding concomitant exposure to specific highly toxic substances and no follow-up throughout the year (Table 1).

The mean age was 36.6 ± 15.7 years, of which 49.0% were male. The 2421 cases were matched to the non-COP group, using a 1:4 ratio based on age and gender, resulting in 9684 COP-free cases in the general population. Based on the general characteristics of all patients, the COP group had significantly higher mental health history, smoking, alcohol consumption, Charlson Comorbidity Index (CCI), mortality, hypertension, diabetes, long-term CVD incidence, and acute kidney injury compared with the non-COP group.

### 3.2. Primary Outcomes Incident Long-Term CVD and DNS/PNS with Mortality as Secondary Outcome

To validate the potential effects of CVD, 10,695 of the COP patients excluding CVD history were analyzed as a subgroup analysis to compare the long-term CVD risk between the COP group and the general population (Table 2). After excluding prior CVD cases in COP patients, there were still 2139 cases, of which 162 still had CVD (7.6%). The average age of this group was 33.7 ± 13.0 years, with 48.6% male. Based on age and sex, a ratio of 1:4 was used to randomly match 8556 individuals from the general population who were not poisoned to serve as a non-COP group. There were significant differences in the basic characteristics between the two groups, except for diabetes and high cholesterol, which were no significant difference. The mortality for the COP patients was 6.6%, which is higher than the 1.9% in the non-COP group. In addition, the COP group presented a greater prevalence of mental illness, smoking, alcohol use, CCI, high blood pressure, and acute kidney injury.

### 3.3. Subgroup Analysis: Risk Indicators of CVD in COP Patients Without Prior CVD History

Regardless of whether the COP patients had CVD history or not, they were still treated according to the actual treatment, but there were no significant differences in these treatment conditions (Table 3). However, COP patients including CVD history had a higher incidence of PNS in group I by 8.6% (*p* < 0.001) and group II by 11.5% (*p* = 0.018), but COP patients who excluded CVD history had a higher incidence of DNS only in group II by 6.8% (*p* < 0.001).

### 3.4. Risk Indicators of CVD in COP Patients Without Prior CVD History

**Figure 1:**
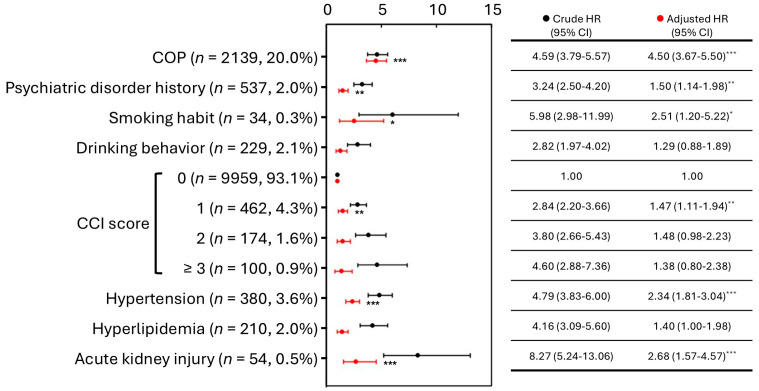
Subgroup analysis of incident long-term CVD risk occurring > 1 year after the index event in COP patients excluding pre-index CVD history (n = 10,695). Abbreviations: CVD, cardiovascular disease; COP, carbon monoxide poisoning; CCI, Charlson Comorbidity Index; HR, hazard ratio; CI, confidence interval. *, *p* < 0.05; **, *p* < 0.01; ***, *p* < 0.001.

The correlation between COP and the subsequent CVD risk was analyzed for a subgroup analysis. After adjusting for other confounding factors, the developing CVD risk in the COP group was found to be 4.50 times (Figure 1). In addition, other relevant risk factors, including mental illness, smoking habits, CCI, high blood pressure, and acute kidney injury, also influenced the COP patients without prior CVD history.

**Figure 2:**
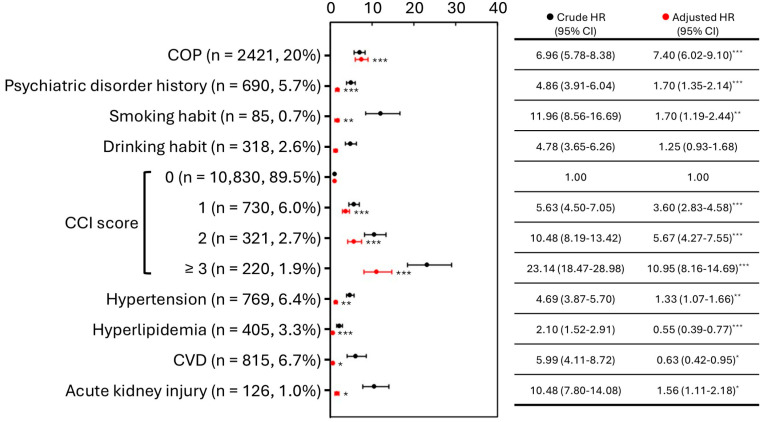
Association estimates for all-cause mortality following COP in Taiwan, 2000–2015 (n = 12,105). Abbreviations: COP, carbon monoxide poisoning; CCI, Charlson Comorbidity Index; CVD, cardiovascular disease; HR, hazard ratio; CI, confidence interval. *, *p* < 0.05; **, *p* < 0.01; ***, *p* < 0.001.

Furthermore, an analysis was also performed on these 12,105 cases to analyze the correlation between COP and subsequent long-term mortality risk (Figure 2). Before adjusting for confounding factors, the long-term mortality risk for those with COP was 6.96 times that of the general population. After controlling other confounding factors, this risk was even more pronounced, being 7.40 times greater than that of the general population (*p* < 0.001). In addition, there are still some higher significant differences in terms of risk factors associated with long-term mortality, including history of mental illness, smoking behavior, CCI, hypertension, and acute kidney injury. In contrast, the adjusted HR for hyperlipidemia and CVD was significantly less than 1.

### 3.5. The Impact of Mental Factors on COP Patients

**Figure 3:**
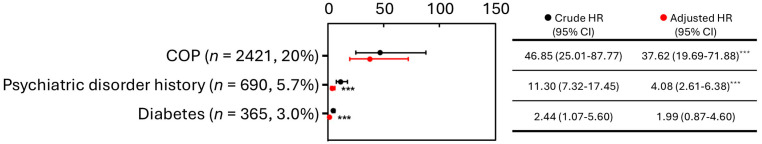
Association estimates for suicide mortality following COP in Taiwan, 2000–2015 (n = 12,105). Abbreviations: COP, carbon monoxide poisoning; HR, hazard ratio; CI, confidence interval. ***, *p* < 0.001.

When analyzing specific causes of death and psychiatric disorder, the suicide mortality in the COP group was 3.1%, while the CVD mortality was 0.6% (Table 1). Both rates were higher than those in the non-COP group, which were 0.1% for suicide and 0.3% for CVD. In addition, the rate of psychiatric disorder history in the COP group was 16.0%, higher than the 3.1% in the non-COP group (*p* < 0.001). An analysis was performed on these 12,105 cases to analyze the correlation between COP and subsequent long-term mortality risk. After adjusting for confounding factors, the aHR of all causes, suicide, and CVD are 7.40, 37.62, and 11.92 respectively; note suicide as a competing event (Figure 3).

**Figure 4:**
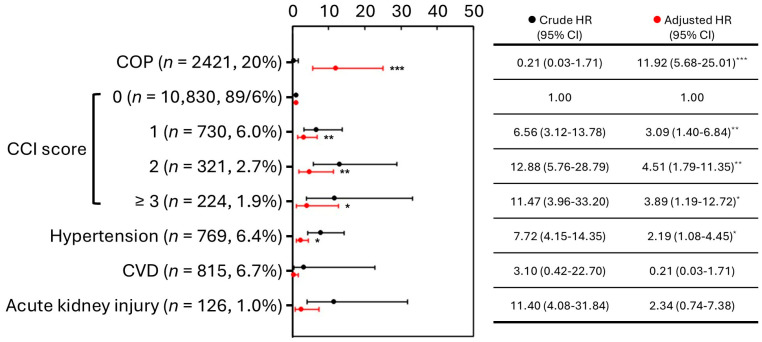
Association estimates for CVD-related mortality following COP in Taiwan, 2000–2015 (n = 12,105). Abbreviations: CVD, cardiovascular disease; COP, carbon monoxide poisoning; CCI, Charlson Comorbidity Index; HR, hazard ratio; CI, confidence interval. *, *p* < 0.05; **, *p* < 0.01; ***, *p* < 0.001.

While there was no evident difference between the subsequent risk of death from CVD risk in COP patients and the general population before adjusting for other confounding factors, after making the adjustments, the risk of dying from CVD following COP was found to be 11.92 times higher than that of the general population (*p* < 0.001) (Figure 4). The associated risk factors for this outcome clearly included CCI and hypertension. In contrast, the adjusted HR for CVD was still less than 1.

### 3.6. Long-Term Neurological Complications in COP Patients

Based on neurological complications, the 2421 patients were categorized into two groups of diagnostic codes to determine DNS or PNS (Table 4). By using group I of diagnostic codes, 55 patients were potential DNS cases, 208 were potential PNS cases, and 2158 did not develop either. A basic comparison of characteristics across these groups revealed that the group without PNS or DNS development had younger, lower rates of mental illness history, lower CCI, and fewer metabolic syndrome diseases. The DNS group had a mortality risk of 0.44 times that of the non-COP group.

In contrast, by using group II of diagnostic codes, the DNS group had a mortality risk of 0.90 times that of the non-COP group; the PNS group had a mortality risk of 1.16 times that of the non-COP group. However, none of these differences reached statistical significance (Table 4).

Regarding deaths from CVD, the risk of cardiovascular mortality was 3.10 times higher in the PNS group using the first group of ICD-9 codes than in the non-COP group, a statistically significant difference. For the second group of ICD-9 codes, although not statistically significant, the risk was also higher, at 2.10 times that of the non-COP group (Table 5).

## 4. Discussion

Using data on the COP patients collected by the Taiwan Poison Control Center (PCC), the development of DNS in COP patients is primarily associated with factors such as age, a history of mental illness, lower coma scores, and myocardial injury [17,18]. Some studies have shown that in the United States, the leading causes of death among poisoned people are neurological and psychiatric disorders, injuries, and violence [19,20]. Here, the NHIRD was utilized to analyze and compare the long-term mortality and CVD risk between patients with COP and general population. The mortality for 2421 COP patients was 8.8%, which is higher than 3.7% in the non-COP group (Table 1). After adjusting for confounding factors, the risk of death remained as high as 7.40 times (Figure 2).

In 2006, a study focusing on cardiovascular outcomes, using indicators like left ventricle ejection fraction to investigate the effects of COP on heart health, concluded that COP leads to CVD risk [1]. However, most patients were able to recover from cardiac functional impairment within 24 h after COP, though a minority sustained CVD risk over a longer term. Moreover, another study by Henry et al. showed that patients at risk for CVD at the initial stages of COP had significantly higher subsequent mortality compared to those without CVD risk [3]. They showed that 32 of the 85 individuals who displayed signs of cardiac distress passed away during an average observation period of 8 years, but 22 out of 145 individuals who did not show cardiac distress passed away (with an adjusted HR of 2.10). Further historical cohort studies have indicated a link between COP and the emergence of critical cardiovascular incidents (adjusted HR of 2.00) [21]. Here, the NHIRD was utilized to investigate the impact on CVD risk after COP, the results were consistent with the past literature. Specifically, COP patients had a significantly higher ratio of developing CVD compared to the general population (Table 1 and Table 2). Additionally, the death risk from CVD in COP patients was also significantly higher.

The detailed clinical data on whether they develop DNS or PNS after COP were not included in the NHIRD. According to the underlying diagnostic codes and time intervals since the COP event, relying solely on diagnostic codes may lead to underestimations or overestimations of incidence. To avoid potential misclassification due to underestimation (which could lead to insufficient sample sizes for analysis) or overestimation, two groups of diagnostic codes with different coverage scopes for categorization (Table 3, Table 4 and Table 5). The results indicated that the outcomes from the two definitions were similar, and there was no significant difference in the risk of long-term mortality between the prognostic groups of DNS patients in group I, including or excluding CVD history (Table 3). The higher DNS incidence in patients without prior CVD was observed only under group II, suggesting that this subgroup finding was definition-sensitive and should be interpreted cautiously. Regarding the prognosis of COP, some biochemical markers were identified in South Korea as potentially important indicators for long-term post-COP outcomes [22,23]. In addition, acute kidney injury following poisoning might lead to poorer outcomes, such as neurological impairments within 28 days post-COP [24]. Due to data collection limitations, efforts should be made to gather more comprehensive cardiac and renal function indicators in COP patients to identify key factors that might influence their prognosis. The COP group had a higher history of mental illness (16.0%), which was another risk factor (4.08 times, *p* < 0.001) (Figure 3). The risk of death from CVD was up to 11.92 times in the COP patients, including CVD history (Figure 4). In contrast, there was no difference in the COP patients, excluding CVD history (Figure 1). Even though, CVD history resulted in a higher incidence of PNS in both groups, but a higher incidence of DNS only in group II (Table 3). This suggests that CVD history is still a potential factor in the development of neurological sequelae, particularly PNS.

There were some limitations in this study. First, the NHIRD lacked crucial clinical data such as the cause of COP, exposure duration, laboratory results (including carboxyhemoglobin and cardiac injury biomarkers such as troponin), vital signs, and the presence of DNS, which prevented precise assessment of the COP severity, time to medical care, or identification of unintentional carbon monoxide exposure. We therefore used endotracheal intubation and intensive care unit admission as pragmatic proxies for severe COP. Future studies linking claim data with hospital electronic medical records or poison registries are needed to incorporate carboxyhemoglobin and troponin measurements. COP is based on patients receiving hyperbaric oxygen therapy or hospitalization after an emergency visit, potentially excluding those with mild poisoning and leading to selection bias. DNS diagnosis relies on physician observations, and using diagnostic codes might under or overestimate DNS incidence. Despite the efforts to address this with various definitions, misclassification may still occur, and the small number of DNS/PNS cases might affect validity. Second, suicide is an important competing event after COP. Using a cause-specific Cox approach, competing deaths were treated as censoring; thus, our hazard ratios describe relative associations rather than cumulative incidence. Future studies using competing-risk methods (e.g., Fine–Gray) may better quantify absolute risk. Third, the dataset does not include specific information to distinguish between intentional and unintentional COP causes, which is a limitation of using the NHIRD for this study. However, this research aims to explore the potential COP effects, specifically investigating the risk factors associated with the development of CVD risk and neurological sequelae in patients affected by carbon monoxide. Importantly, the study outcomes are not affected by the distinction between intentional and unintentional cases. Fourth, PCC provides comprehensive exposure data and detailed clinical examination and treatment records, but it lacks information on the long-term prognosis of patients. If a patient is lost to follow-up or seeks care at a different institution, collecting pertinent data becomes challenging. As NHIRD is a national database, it provides extensive coverage and integration of patient records across various institutions, ensuring that the dataset is extensive and representative, and therefore does not compromise the conclusions here.

Because of budget constraints, we only had data available through 2015. Furthermore, Taiwan’s NHI claims transitioned from ICD-9-CM to ICD-10-CM in 2016 [25]. Validation of ICD-10-CM varies across diseases. COP and acute myocardial injury both have good diagnostic validity in ICD-10-CM, but the identification of valvular heart disease may be inconsistent across ICD-10-CM algorithms during the ICD-9-to-ICD-10 transition [26,27,28]. These differences in validation limited the use of ICD-10-CM in our article, compared with ICD-9-CM. Regarding generalizability, because our COP definition relied on inpatient claims and treatment-related criteria (e.g., oxygen/HBO therapy), the results might be less generalizable to milder or non-hospitalized CO exposure. In addition, our inclusion period ended in 2015, and mortality linkage was available through 2015. Extrapolation to the post-2016 ICD-10 era should be made cautiously. Finally, because long-term CVD outcomes were defined as occurring more than one year after COP, individuals poisoned in 2015 had inherently limited time for long-term follow-up, which might widen confidence intervals and affect temporal generalizability for the most recent entry year.

## 5. Conclusions

COP patients had a higher long-term neurological complication than the general population, especially from CVD risk and suicide. Future studies are warranted to develop a reliable prediction model for CVD risk and PNS, which may assist clinicians in decision-making and follow-up planning.

## Figures and Tables

**Table 1:** Characteristics and follow-up outcomes of COP patients and age- and sex-matched non-COP controls in Taiwan, 2000–2015. (n = 12,105).

Variables		COP	Non-COP	*p* Value
Case no.		2421	9684	
Age (years), mean ± SD		36.6 ± 15.7	36.6 ± 15.7	-
Male gender, *n* (%)		1186 (49.0%)	4744 (49.0%)	-
Psychiatric disorder history, *n* (%)		388 (16.0%)	302 (3.1%)	<0.001
Smoking habit, *n* (%)		28 (1.2%)	57 (0.6%)	0.003
Drinking behavior, *n* (%)		100 (4.1%)	218 (2.3%)	<0.001
CCI score, *n* (%)				<0.001
0		2098 (86.7%)	8732 (90.2%)	
1		176 (7.3%)	554 (5.7%)	
2		78 (3.2%)	243 (2.5%)	
≥3		69 (2.9%)	155 (1.6%)	
Death, *n* (%)	Total	212 (8.8%)	363 (3.7%)	<0.001
Suicide		76 (3.1%)	13 (0.1%)	
CVD		15 (0.6%)	30 (0.3%)	
Others		121 (5.0%)	320 (3.3%)	
Hypertension, *n* (%)		189 (7.8%)	580 (6.0%)	0.001
Diabetes, *n* (%)		88 (3.6%)	277 (2.9%)	0.046
Hyperlipidemia, *n* (%)		89 (3.7%)	316 (3.3%)	0.312
CVD, *n* (%)		195 (8.1%)	620 (6.4%)	0.004
Acute kidney injury, *n* (%)		38 (1.6%)	88 (0.9%)	0.004

Abbreviations: COP, carbon monoxide poisoning; SD, standard deviation; CCI, Charlson Comorbidity Index; CVD, cardiovascular disease.

**Table 2:** Characteristics of COP patients, excluding pre-index CVD history, for subgroup analysis of incident long-term CVD occurring > 1 year after the index event (n = 10,695).

Variables		COP	Non-COP	*p* Value
Case no.		2139	8556	
Age (years), mean ± SD		33.7 ± 13.0	33.7 ± 13.0	-
Male gender, *n* (%)		1039 (48.6%)	4156 (48.6%)	-
Psychiatric disorder history, *n* (%)		318 (14.9%)	219 (2.6%)	<0.001
Smoking habit, *n* (%)		16 (0.8%)	18 (0.2%)	<0.001
Drinking behavior, *n* (%)		79 (3.7%)	150 (1.8%)	<0.001
CCI score, *n* (%)				0.001
0		1951 (91.2%)	8008 (93.6%)	
1		119 (5.6%)	343 (4.0%)	
2		42 (2.0%)	132 (1.5%)	
≥3		27 (1.3%)	73 (0.9%)	
Death, *n* (%)	Total	142 (6.6%)	160 (1.9%)	
Suicide		67 (3.1%)	10 (0.1%)	
CVD		5 (0.2%)	10 (0.1%)	
Others		70 (3.3%)	140 (1.6%)	
Hypertension, *n* (%)		93 (4.4%)	287 (3.4%)	0.026
Diabetes, *n* (%)		43 (2.0%)	141 (1.7%)	0.249
Hyperlipidemia, *n* (%)		48 (2.2%)	162 (1.9%)	0.296
CVD, *n* (%)		162 (7.6%)	487 (5.7%)	0.001
Acute kidney injury, *n* (%)		20 (0.9%)	34 (0.4%)	0.002

Abbreviations: COP, carbon monoxide poisoning; CVD, cardiovascular disease; SD: standard deviation; CCI, Charlson Comorbidity Index.

**Table 3:** Treatment and neuropsychiatric outcomes of COP patients stratified by CVD history, defining PNS up to 6 weeks and DNS within 3 months after completion of treatment.

Variables	CVD History		*p* Value
	COP Including	COP Excluding	
Case no.	2421	2139	
Concomitant use of tranquilizer, *n* (%)	165 (6.8%)	145 (6.8%)	1.000
Endotracheal intubation, *n* (%)	212 (8.8%)	172 (8.0%)	0.456
HBO therapy, *n* (%)	2338 (96.6%)	2071 (96.8%)	0.966
Sessions of HBO, mean ± SD	5.2 ± 11.4	5.1 ± 10.7	0.761
Length of hospitalization (day), mean ± SD	0.7 ± 10.7	0.6 ± 11.2	0.758
ICU stay, *n* (%)	353 (14.6%)	308 (14.4%)	0.901
DNS1 ^a^, *n* (%)	55 (2.3%)	45 (2.1%)	0.762
PNS1 ^a^, *n* (%)	208 (8.6%)	70 (3.3%)	<0.001
DNS2 ^b^, *n* (%)	79 (3.3%)	146 (6.8%)	<0.001
PNS2 ^b^, *n* (%)	279 (11.5%)	195 (9.1%)	0.018

^a^ group I by DNS1/PNS1 codes: 438, 348.1, 348.9, 799.0, 290.1, 294.1, 294.8, 294.9. ^b^ group II by DNS2/PNS2 codes: 438, 348.1, 348.9, 799.0, 290.1, 294.1, 294.8, 294.9, 430, 431, 432, 433, 434, 435, 436, 294, 290.0, 290.2, 290.3, 293.0, 293.1, 293.8, 293.9. Abbreviations: COP, carbon monoxide poisoning; CVD, cardiovascular disease; HBO, hyperbaric oxygen; SD: standard deviation; ICU, intensive care unit; DNS, delayed neurological sequelae; PNS, persistent neurological sequelae.

**Table 4:** Association estimates for all-cause death among COP patients with neuropsychiatric sequelae by diagnostic code group, using PNS up to 6 weeks and DNS within 3 months after completion of treatment (n = 2421).

Variables	Case (%)	Adjusted HR (95% CI)	
		Group I	Group II
DNS1 ^a^, *n* (%)	55 (2.3%)	0.44 (0.17–1.09)	
PNS1 ^a^, *n* (%)	208 (8.6%)	1.11 (0.77–1.58)	
DNS2 ^b^, *n* (%)	79 (3.3%)		0.90 (0.45–1.79)
PNS2 ^b^, *n* (%)	279 (11.5%)		1.16 (0.84–1.61)
Age (years), mean ± SD	36.6 ± 15.7	1.04 (1.03–1.05) ***	1.04 (1.03–1.05) ***
Male gender, *n* (%)	1186 (49.0%)	0.49 (0.37–0.66) ***	0.49 (0.37–0.66) ***
Psychiatric disorder history, *n* (%)	388 (16.0%)	2.28 (1.67–3.12) ***	2.25 (1.65–3.07) ***
Smoking habit, *n* (%)	28 (1.2%)	1.92 (0.99–3.71)	1.75 (0.91–3.38)
Drinking behavior, *n* (%)	100 (4.1%)	1.99 (1.25–3.18) **	1.98 (1.24–3.17) **
CCI score, *n* (%)			
0	2098 (86.7%)		
1	176 (7.3%)	1.88 (1.25–2.84) **	1.88 (1.24–2.85) **
2	78 (3.2%)	2.25 (1.36–3.72) **	2.19 (1.33–3.60) **
≥3	29 (1.2%)	1.80 (1.01–3.21) *	1.92 (1.08–3.43) *
Hyperlipidemia, *n* (%)	405 (16.7%)	0.49 (0.25–0.93) *	0.48 (0.25–0.93) *
Acute kidney injury, *n* (%)	126 (5.2%)	2.26 (1.23–4.17) **	1.93 (1.06–3.52) *
Endotracheal intubation, *n* (%)	212 (8.8%)	1.98 (1.39–2.82) **	1.91 (1.35–2.72) ***
CVD, *n* (%)	815 (33.7%)	0.76 (0.50–1.15)	

*, *p* < 0.05; **, *p* < 0.01; ***, *p* < 0.001. ^a^ group I by DNS1/PNS1 codes: 438, 348.1, 348.9, 799.0, 290.1, 294.1, 294.8, 294.9. ^b^ group II by DNS2/PNS2 codes: 438, 348.1, 348.9, 799.0, 290.1, 294.1, 294.8, 294.9, 430, 431, 432, 433, 434, 435, 436, 294, 290.0, 290.2, 290.3, 293.0, 293.1, 293.8, 293.9. Abbreviations: DNS, delayed neurological sequelae; PNS, persistent neurological sequelae; HR, hazard ratio; CI, confidence interval; SD, standard deviation; CCI, Charlson Comorbidity Index; CVD, cardiovascular disease.

**Table 5:** Association estimates for CVD-related death among COP patients with neuropsychiatric sequelae by diagnostic code group, using PNS up to 6 weeks and DNS within 3 months after completion of treatment. (n = 2421).

Variables	Case (%)	Adjusted HR (95% CI)	
		Group I	Group II
DNS1 ^a^, *n* (%)	55 (2.3%)	0 (0–.)	
PNS1 ^a^, *n* (%)	208 (8.6%)	3.10 (1.07–8.99) *	
DNS2 ^b^, *n* (%)	79 (3.3%)		0 (0-.)
PNS2 ^b^, *n* (%)	279 (11.5%)		2.10 (0.73–6.02)
Age (years), mean ± SD	36.6 ± 15.7	1.07 (1.04–1.10) ***	1.07 (1.04–1.10) ***
Endotracheal intubation, *n* (%)	212 (8.8%)	2.72 (0.86–8.63)	3.10 (0.98–9.80)

*, *p* < 0.05; ***, *p* < 0.001. ^a^ group I by DNS1/PNS1 codes: 438, 348.1, 348.9, 799.0, 290.1, 294.1, 294.8, 294.9. ^b^ group II by DNS2/PNS2 codes: 438, 348.1, 348.9, 799.0, 290.1, 294.1, 294.8, 294.9, 430, 431, 432, 433, 434, 435, 436, 294, 290.0, 290.2, 290.3, 293.0, 293.1, 293.8, 293.9. Abbreviations: CVD, cardiovascular disease; DNS, delayed neurological sequelae; PNS, persistent neurological sequelae; SD, standard deviation; HR, hazard ratio; CI, confidence interval.

## Data Availability

The data underlying this study are from the National Health Insurance Research Database (NHIRD), which has been transferred to the Health and Welfare Data Science Center (HWDC). The NHIRD is not free to public access, and therefore interested researchers can obtain the data through formal application to the HWDC, Department of Statistics, Ministry of Health and Welfare, Taiwan (https://dep.mohw.gov.tw/DOS/cp-5119-59201-113.html, accessed on 2 February 2026). The authors had no special access privileges that others would not have.
